# WI-38 senescence is associated with global and site-specific hypomethylation

**DOI:** 10.18632/aging.100679

**Published:** 2014-07-19

**Authors:** Corinne Sidler, Rafal Woycicki, Igor Kovalchuk, Olga Kovalchuk

**Affiliations:** Department of Biological Sciences, University of Lethbridge, Lethbridge, AB, T1K 3M4, Canada

**Keywords:** WI-38, senescence, aging, DNA methylation, hypomethylation, Illumina HumanMethylation27 BeadChip

## Abstract

Cellular senescence plays an important role in the age-dependent functional decline of organs and organ systems, as well as in age-related pathologies, such as cancer. Therefore, a better understanding of its underlying molecular mechanisms is crucial in the search for intervening measures. In this study, we considered the role of DNA methylation in senescence. We found that senescence is associated with global DNA hypomethylation, but also involves site-specific DNA hypo- and hypermethylation. In some cases, this differential methylation may affect gene expression and thereby modulate functional processes within cells. However, the majority of the CpG sites that were differentially methylated did not correspond with altered gene expression, suggesting that DNA methylation affects senescence by other means also, such as, for instance, genome stability.

## INTRODUCTION

Cellular senescence is associated with the terminal cell cycle arrest of cells, and was first described by Hayflick and Moorhead, who observed that human diploid fibroblasts exhibit a limited *in vitro* replicative lifespan of approximately 50 ± 10 population doublings, after which the cell cultures progressively decline [1, 2]. Later, such senescent cells were shown to accumulate in tissues *in vivo* with increasing age [3, 4], and when primary cells from species with different maximum lifespans were taken into a cell culture, their *in vitro* lifespan correlated with the maximum lifespan of the donor species [5, 6]. These observations supported a role for cellular senescence in organismal aging.

There are several different ways, in which cellular senescence may contribute to the age-related functional decline of organs and organ systems and age-related pathologies. On the one hand, the senescence of cells that rely on proliferation for their proper function, such as stem cells and immune cells, limits the repair capacity in aging tissue [7] and contributes to the age-dependent deterioration of the immune system [8]. On the other hand, while the pro-inflammatory micro-environment of senescent cells can contribute to the development and progression of inflammatory diseases and cancer [9, 10], the failure of senescence mechanisms can also result in malignant transformation and cancer progression [11]. Further, a systems biology approach showed the similarity between genes involved in cellular senescence, aging and age-related pathologies, suggesting these may have co-evolved [12]. Thus, understanding the mechanisms that underlie senescence constitutes a major step towards the goal of ensuring healthy aging.

While certain molecular changes have already been associated with the induction of senescence, such as telomere shortening [13, 14], and the increased expression of senescence-associated genes like *CCND1* [15, 16], the role of epigenetic phenomena [17] and changes in chromatin structure have recently also been considered for their roles in aging and senescence (see also [18-20]). Narita et al. [21] were the first to describe the focal formation of heterochromatin in senescent cells, which they termed “senescence-associated heterochromatin foci” (SAHF). They showed that this heterochromatin formation was targeted to E2F target genes by RB/E2F and reinforced terminal cell cycle arrest through the permanent downregulation of cell cycle regulators. Swanson et al. [22], meanwhile, described an early step in the establishment of senescence that they termed the “senescence-associated distension of satellites” (SADS), which was caused by the relaxation of the chromatin structure in pericentromeric satellite regions. Thus, both the loss of heterochromatin in regions of constitutive heterochromatin and the gain of heterochromatin in previously transcriptionally active regions may contribute to the establishment of senescence.

Several recent studies involved massive parallel sequencing projects that compared the DNA methylomes of blood cells from donors of varying ages ranging from newborns to centenarians [23-25]. They commonly observed a decrease in global DNA methylation with increasing age. This loss of DNA methylation was observed in diverse regions of the genome and affected various functional groups of genes. Heyn et al. (2012) found that mainly CpG-poor and tissue-specific promoters were affected by DNA hypomethylation, while McClay et al. (2014) observed reduced DNA methylation levels in regions associated with polycomb proteins or activating histone marks. DNA hypermethylation, on the other hand, occurred more frequently in CpG-rich sequences, and seemed to be more site-specific than DNA hypomethylation. In addition, a comparison of the DNA methylomes of different cell types at different passages showed that the differential methylation of several sites served as a biomarker for senescence [26].

Even though DNA hypomethylation seemed to be less site-specific than DNA hypermethylation, the observations that the loss of DNA methylation only occurs in senescing cell strains, and not in immortalized cells [27], and that the inhibition of DNA methylation can induce growth arrest in immortal cells [28], suggest that DNA hypomethylation does play an important role in the establishment of senescence.

Here, we investigate the role of differential DNA methylation in senescence in WI-38 human diploid lung fibroblasts. Senescent WI-38 cells exhibited down-regulation of DNMT1 and reduced global DNA methylation. The extensive changes to the DNA methylation profile are shown to affect partially distinct functional groups of genes according to whether there is hypomethylation or hypermethylation. However, changes in DNA methylation only corresponded with differential gene expression in a small fraction of the affected sites.

## RESULTS

### Senescence is associated with reduced global DNA methylation

**Figure 1:**
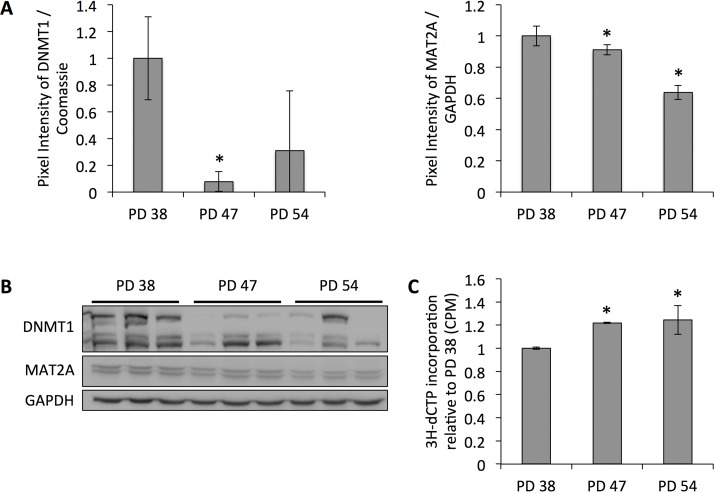
Reduced protein levels of DNMT1 correlate with decreased global DNA methylation (**A**) DNMT1 and MAT2A protein levels normalized to protein levels in PD 38 cells. Averages of three biological replicates ± standard deviation. (**B**) Western blot images. (**C**) Cytosine extension assay showing average incorporation of radioactively labeled dCTP from three samples per PD level; error bars indicate standard.

To date, several studies have shown age-dependent alterations in the DNA methylation profile of cells. While both hypermethylated and hypomethylated sites have been detected with the increasing age of the subjects, DNA hypomethylation was more frequent [23-25]. Our previous results indicated that DNA methyltransferase *DNMT1*, which performs maintenance and *de novo* DNA methylation and is the predominant DNA methyltransferase in somatic cells [29, 30], and Methionine adenosyltransferase 2 alpha (*MAT2A* ), which plays a role in the formation of S-adenosylmethionine (SAM), the substrate for DNA methylation, were downregulated in senescent cells (manuscript 000141R1 accepted in *AGING* ). This was confirmed at the protein level (Fig. 1A, B) and correlated with decreased global DNA methylation, as shown by the increased incorporation of radioactively labeled dCTP into DNA from senescent cells (Fig. 1C).

### Hypomethylation of CpG sites in the promoter regions of genes is observed with increasing senescence ratio of cultures

In order to study how this deregulation of DNMT1 and the global DNA hypomethylation may affect the DNA methylation patterns at specific sites, genomic DNA was extracted from cultures of different PD levels and assessed for the methylation status of CpG sites with regulatory functions in the promoter regions of genes using Illumina® HumanMethylation27 BeadChips, which interrogate 27,578 CpG sites spanning 14,495 genes (for detailed results, see S1).

**Figure 2:**
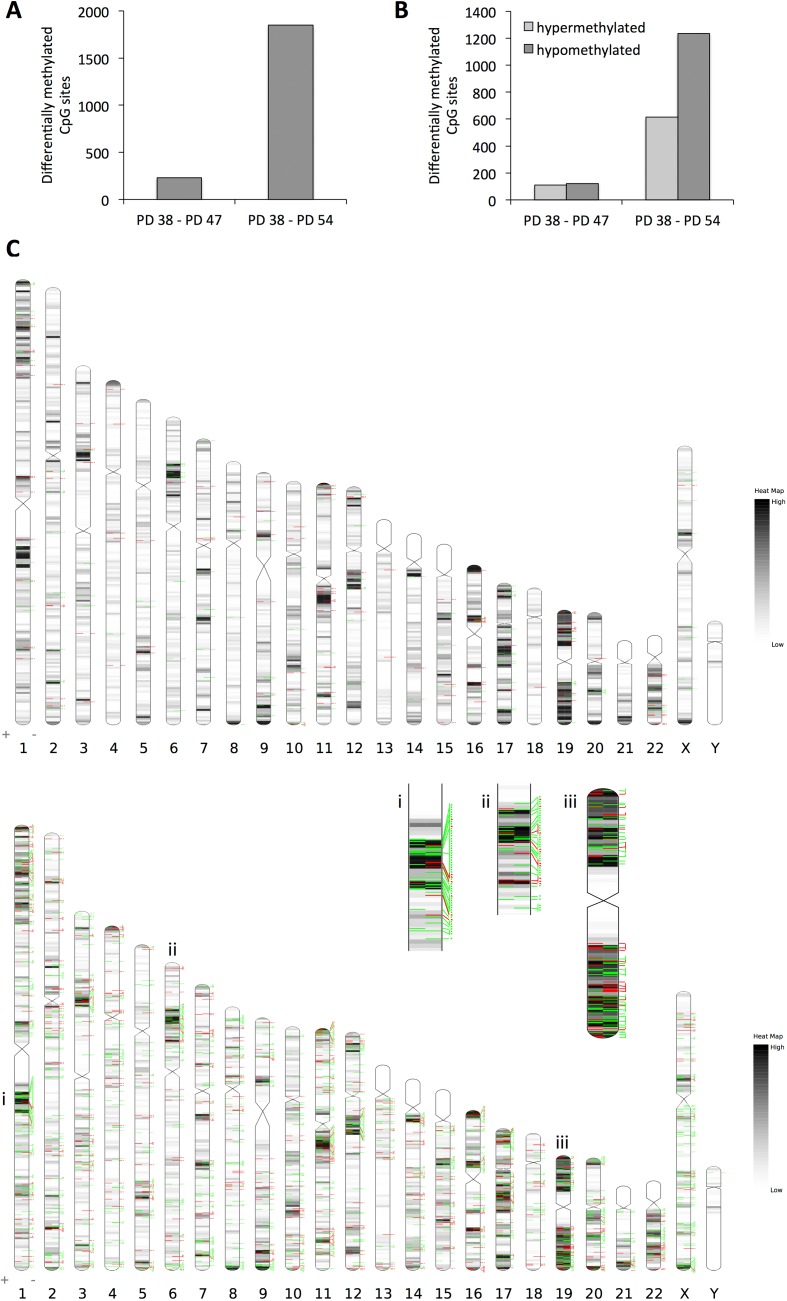
Changes in DNA methylation pattern in senescent cells (**A**) Number of CpG sites affected by differential methylation comparing PD 47 and PD 54 to PD 38 cultures. (**B**) Number of CpG sites in the given comparisons affected by hypo- (dark grey) or hypermethylation (light grey). (**C**) Distribution of CpG methylation changes across the chromosomes. Top: changes between PD 47 and PD 38; Bottom: changes between PD 54 and PD 38. Black/grey lines indicate gene densities, green lines represent CpG sites hypomethylated in the older cultures and red lines indicate CpG sites hypermethylated in older cultures.

When comparing the PD 54 to PD 38 cultures, the array data showed that 1,849 CpG sites were affected by differential methylation (Fig. 2A); these sites were predominantly hypomethylated (66.7% of all differentially methylated sites) (Fig. 2B). This increasing hypomethylation of CpG sites is in line with the observed reduction in *DNMT1* expression and global DNA hypomethylation in more senescent cultures (Fig. 1).

When comparing the PD 47 and PD 38 cultures, the 231 CpG sites affected by differential methylation appear to be randomly distributed across the genome, whereas when comparing PD 54 and PD 38, several clusters with increased incidence of hypomethylated or hyper-methylated CpG sites were found (Fig. 2C). Examples of such clusters are shown in Figure 2C (i, ii, iii). The observed DNA methylation changes were detected predominantly in regions of moderate or high gene density, probably due to the enrichment of probe sets specific to those regions on the BeadChip that was used for this study. Therefore, the absence of DNA methylation changes detected in gene-poor regions of the genome does not allow for any conclusions about changes in the DNA methylation status in those regions. Interestingly, in the PD 54 and PD 38 comparison, only 42 genes that showed differential promoter methylation were also affected by changes in gene expression (Table 1), while none of the genes with differentially methylated promoter regions were found to be differentially expressed when comparing the PD 47 and PD 38 cultures. Among those 42 genes, for 24 genes the change in gene expression corresponded with the change in DNA methylation. These genes included *CDK2* and *E2F2*, both regulators of the G1/S progression, which were transcriptionally repressed and showed hypermethylation in their promoter regions (Table 1). Hypermethylation of the promoters of these genes may mediate the permanent cell cycle arrest of senescent cells in the G1/G0 phase of the cell cycle.

**Table 1:** Genes affected by differential expression and DNA methylation Numbers indicate log2-fold changes in the transcript level or CpG methylation level when comparing PD 54 to PD 38 cultures; information on gene function retrieved from Genecards [53].

	Gene expression	DNA methylation	Gene function
**ALDH1A3**	−0.41	0.78	Aldehyde dehydrogenase, which binds to retinal
**APP**	0.72	−1.11	Amyloid beta (A4) precursor protein, best studied for its role in Alzheimer's disease
**ATP5D**	0.48	1.1	Mitochondrial membrane ATP synthase
**CDK2**	−0.43	0.55	Cyclin-dependent kinase, mediates G1/S progression together with CCNE and the transition from S to G2 phase with CCNA
**COL16A1**	−0.43	−0.68	Type XVI Collagen, involved in mediating cell attachment and morphology
**CPS1**	−0.59	−0.52	Mitochondrial Carbamoyl Phosphate Synthase 1, involved in the urea cycle
**DPP4**	0.46	0.4	Dipeptidyl-Peptidase 4, membrane glycoprotein, serine exopeptidase
**E2F2**	−0.6	1.94	Transcription factor involved in the regulation of G1/S transition and DNA replication
**FHL1**	0.57	−0.42	Contains zinc finger domains and LIM domains, may be involved in muscle development
**GFPT2**	−0.53	2.48	Controls the flux of glucose to the hexosamine pathway
**GPI**	0.64	0.73	Glucose-6-Phosphate Isomerase, involved in Glycolysis
**GSTO2**	−0.45	0.49	Glutathione S-Transferase Omega 2, may be involved in ascorbic acid recycling
**HMGB2**	−1.14	−1.94	Preferentially binds to ssDNA and may bend it, co-factor to RAG during V(D)J recombination
**HYLS1**	−0.7	−0.97	Associated with centriole, involved in cilia formation
**ID3**	1.02	−1.36	Inhibits DNA binding of transcription factors, including E2A
**KIF4A**	−0.72	−0.95	Motor protein, involved in spindle organization
**MLLT11**	0.5	−0.99	Fused with a number of translocation partners in leukemia
**MOV10**	−0.43	−1.53	RNA helicase, involved in RISC-mediated post-transcriptional gene silencing
**MVP**	0.51	0.51	Major vault protein, plays a role in the formation of scaffolds for signal transduction
**NEFH**	−0.51	0.56	Neurofilament protein
**NFE2L3**	−0.76	1.5	Transcription factor, which binds to antioxidant response element in target promoters
**NR4A2**	0.83	−1.65	Nuclear Receptor Subfamily 4 (NURR1), may function as a transcription factor
**NTF3**	−0.61	0.54	Neurotrophin 3, involved in neural survival
**PITX1**	1.43	0.61	Transcriptional regulator, involved in the regulation of development
**PPIL5**	−0.5	1.03	Negative regulator of TNFRSF9 signalling
**RAB11FIP5**	0.4	−2.1	Involved in protein trafficking
**RAMP1**	−0.61	1.16	Membrane protein, co-receptor
**RELB**	−0.44	0.5	Dimerizes with NF-κB to modify its preference for transcription target binding
**RELN**	−0.65	−1.07	Extracellular matrix serine protease
**RPL39L**	−0.62	0.86	Ribosomal protein L39 like
**SCAMP3**	**0.45**	**1.84**	**Involved in post-Golgi recycling pathways and protein trafficking**
**SLC4A4**	0.44	−0.59	Sodium/bicarbonate co-transporter
**SLIT2**	−1.33	−1.95	May regulate cell migration
**SPATA18**	0.55	1.23	Involved in the repair or degradation of damaged mitochondria
**SSRP1**	−0.61	0.45	Part of the FACT complex, which is involved in destabilizing and reassembling nucleosomes during transcription, replication and DNA repair
**TFRC**	−0.51	1.19	Receptor involved in iron uptake
**TGIF2**	−0.52	0.56	Transcriptional repressor of TGFbeta responsive genes, through the recruitment of histone deacetylases
**TMEM35**	0.75	−0.67	Transmembrane protein 35
**TMEM47**	0.64	−1.02	Localized to ER
**TMSL3**	−0.45	−0.41	Unknown function
**TRO**	−0.49	−0.71	Involved in cell adhesion
**ZAK**	0.54	1.57	Stress-activated, involved in JNK and p38 pathways

Further, several genes that function as transcription factors or regulate the activity or target specificity of transcription factors were among the 24 genes with corresponding changes in gene expression, *E2F2*, *FHL1*, which enhances the transcriptional activity of NFAT1c [31], *ID3*, which inhibits several transcription factors of the basic-helix-loop-helix family [32], *NFE2L3*, which was identified as a negative regulator of the antioxidant-response element containing promoters [33], *NR4A2*, which can be transcriptionally induced in response to pro-inflammatory signals [34] and may then induce the expression of additional cytokines through the interaction with NF-κB/p65 [35], *RELB*, which is a transcription factor of the NF-κB/RELB family and represses pro-inflammatory gene expression in fibroblasts [36], and TGIF2, which transcriptionally represses TGFβ target genes [37]. This supports the involvement of multiple transcriptional programs in the regulation of the senescence-associated gene expression profile as well as the establishment of the senescence-associated phenotype.

While 52.3% of all differentially methylated CpG sites were located in CpG islands, among the 42 CpG sites that resided in promoters of differentially expressed genes a higher fraction of sites was located in CpG islands – 87.5% of sites which corresponded with the change in gene expression and 72.2% of sites which did not correspond with the change in gene expression. Additionally, differentially methylated sites that corresponded with a gene expression change were located slightly closer to the transcription start site (average distance of 412 nucleotides) when compared to all differentially methylated CpG sites (average distance of 421 nucleotides), while differentially methylated sites that did not correspond with the observed gene expression change were located slightly more distant (average distance of 495 nucleotides). However, due to large variations in the distance to the transcription start site, these differences were not statistically significant. Further, fifteen out of the 24 cases, in which differential DNA methylation corresponded with differential gene expression, consisted of hypermethylated CpG sites and corresponding repression of the transcript.

Thus, while CpG sites located in CpG islands were more frequently associated with differential gene expression, the majority of the observed changes in DNA methylation did not correlate with changes in gene expression. These changes may not affect CpGs that directly regulate the gene expression, but they may modify the accessibility of the promoter region to transcriptional regulation or affect genome stability.

**Figure 3:**
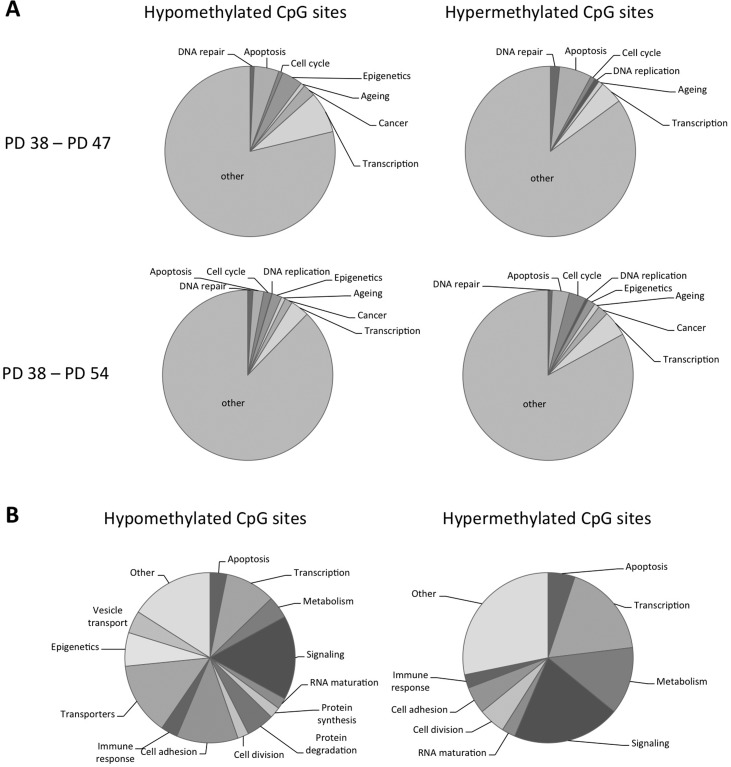
Functional classification of the genes affected by differential DNA methylation (**A**) Pie charts showing functional classification of hyper- and hypomethylated CpG sites. (**B**) Pie charts showing functional classification of CpG sites affected by methylation changes when comparing PD 47 and P38 as well as PD 54 and PD 38 cultures.

In order to better understand the potential functional implications of the differential methylation of all the sites affected, genes with differential methylation status of CpG sites within their promoter regions were functionally classified using the DAVID and FunNet software. This showed that both hypo- and hyper-methylated CpG sites are found in the promoters of genes involved in cell cycle regulation, apoptosis and senescence, as well as in epigenetic and transcriptional regulation (Fig. 3A). When comparing the PD 54 and PD 38 cultures, the remaining genes were enriched for the biological processes of structural development, cell adhesion and localization, transmembrane transport, response to extracellular stimuli and cell signalling and maintenance of cellular homeostasis and ion homeostasis as determined using the g:Profiler software.

When comparing the sites that were differentially methylated between PD 47 and PD 38 to those differentially methylated between PD 54 and PD 38, 52.6% of the sites affected in the PD 47 and PD 38 comparison were found to be common. This may indicate that those sites are subject to differential methylation early during differentiation or the establishment of senescence. To better understand what processes may be affected by differential methylation in both PD 47 and PD 54 cultures, detailed functions of the affected genes were gathered from Genecards and functionally classified (Fig. 3B). Among the genes with hypomethylated CpG sites in their promoter, involvement in transcriptional or epigenetic regulation, cell signalling, cell adhesion and transport of ions were prevalent, whereas among the genes with hyper-methylated promoters, the major functional groups affected were cell signalling, transcriptional regulation and cell metabolism. This resembles the functional groups affected by early gene expression changes and supports the conclusion that transcriptional and epigenetic regulation may affect the establishment of senescence at the level of gene expression (manuscript 000141R1 in *AGING*). Changes in cellular signalling may also be involved in the establishment of senescence by modifying the activation of transcriptional programs in response to extracellular and intracellular stimuli, such as oxidative stress and a pro-inflammatory microenvironment, as well as by inducing changes in the cell physiology.

## DISCUSSION

As reported (manuscript 000141R1 accepted in *AGING*), our previous study indicated that in addition to the numerous transcription factors, 2.7% of all the differentially expressed genes found when comparing PD 54 and PD 38 were associated with functions in epigenetic regulation. Among those, *DNMT1* was downregulated in senescent cells, which correlated with the global reduction of DNA methylation. This supports the conclusion that there is a senescence-dependent loss of heterochromatin, as also observed in different tissues of aging model organisms [25, 27, 38-40]. A study by Fairweather et al. (1987) showed the treatment of human foetal lung fibroblasts (MRC5) with 5-Azacytidine to result in a reduction of the content of methylated CpGs in the genomes of the cells and correlate with a reduced *in vitro* lifespan, thus suggesting that DNA hypomethylation may play an important role in the control of the lifespan of human diploid fibroblasts [41]. This effect may be mediated by loss of DNA methylation in promoter regions resulting in the deregulation of gene expression profiles, as proposed in the heterochromatin-loss model of aging [42]. On the other hand, the loss of DNA methylation in intergenic regions and at repetitive sequences may result in genomic instability through a loss of silencing of those regions [43].

When we considered site-specific differences in DNA methylation when comparing senescent and proliferatively active cell cultures, we found extensive differential methylation of CpGs in the promoter regions of genes (Fig. 3). Two thirds of those sites were affected by hypomethylation, while one third of the sites were hypermethylated. These observations are in line with the finding that aging is associated with a global loss of DNA methylation [25]. Heyn et al. (2012) further showed that differentially methylated CpG sites mostly occurred in the intergenic and intronic regions, with only 10% affecting promoter regions [25]. As the Illumina^®^ HumanMethylation27 BeadChip contains probes that detect methylated CpGs in promoter regions, our dataset may only represent a fraction of the differential CpG methylation associated with senescence in WI-38 cells.

Since the probes on the BeadChip mainly covered CpGs in promoter regions, it is not surprising that only limited events of differential methylation were detected in gene-poor regions (Fig. 3). However, when comparing the DNA methylation patterns in PD 54 to PD 38 cells, some chromosomal regions with clustered events of CpG hypo- or hypermethylation became apparent.

Surprisingly the overlap between the DNA methylation profiling and gene expression profiling datasets is very limited. Only 24 out of the 1,849 differentially methylated CpG sites corresponded with the differential expression of the according gene. This indicates that in this model, DNA methylation changes are not the major regulatory factor of the senescence-associated deregulation of transcription, which is in contrast to the proposed heterochromatin-loss model of aging [42]. However, there were several transcription factors among the genes for which differential DNA methylation correlated with differential gene expression, as well as *CDK2* and *E2F2*, two central regulators of cell cycle progression. These genes exhibited promoter hypermethylation along with reduced transcript expression, indicating that the hard-wiring of the transcriptional repression through DNA methylation may play a role in the maintenance of the permanent growth arrest. The observation that 15 out of the 24 genes were affected by promoter hyper-methylation and transcriptional repression, including *CDK2*, *E2F2*, *TGIF2*, *RELB* and *NFE2L3*, suggests that targeted promoter hypermethylation may affect the regulation of the gene expression profiles to a greater extent than the loss of DNA methylation through inducing a more permanent suppression of the expression of cell cycle and transcriptional regulators. This is in line with the observation that DNA hypermethylation seems to be more targeted than DNA hypomethylation [24].

The results of the functional classification of the genes affected by differential promoter methylation indicated that the processes affected were more diverse than those observed for the changes in gene expression. The differentially methylated promoters when comparing PD 54 and PD 38 showed enrichment in KEGG pathways involved in metabolism (arachidonic acid, glycine/serine/threonine, linoleic acid, nitrogen and glycerolipid metabolism and steroid hormone biosynthesis) and cell signalling (Cytokine-cytokine receptor interaction, NOD-like receptor signalling, JAK-STAT signalling, and Toll-like receptor signalling), while functions in the processes of cell cycle, DNA repair and transcriptional and epigenetic regulation contributed smaller fractions of differentially methylated CpG sites. The overrepresentation of those metabolic and cell signalling pathways corresponds with previously reported changes in lipid metabolism [44] and in the expression of cytokines and TLRs in several aging non immune system cells [45, 46]. Thus, promoter hypomethylation in those cases may increase the accessibility for transcriptional regulators without inducing permanent transcriptional activation, as no changes in transcript levels were detected.

When comparing the sites that were affected by differential methylation between PD 47 and PD 38 to those for PD 54 and PD 38, 52.6% of the PD 47-to-PD 38 differentially methylated CpG sites were found to be consistently differentially methylated. The hyper-methylated CpGs among the overlapping sites mainly affected genes with functions in transcription, metabolism or cell signalling, while the hypomethylated CpGs affected a higher variety of functional groups, including transcriptional regulation, cell signalling, cell adhesion, transporters and epigenetic regulators.

Further, this 52.6% overlap in differential methylation is considerably smaller than the 80% overlap observed for the gene expression profiles (manuscript 000141R1 accepted in *AGING*), suggesting that changes in DNA methylation may be more dynamic across the cellular lifespan. In addition, several of our findings indicate a role of DNA methylation in the establishment of senescence that does not solely depend on the regulation of gene expression - the majority of the differentially methylated CpG sites when comparing PD 54 and PD 38 cultures were hypomethylated, while the majority of differentially expressed genes were downregulated, and only 24 out of 1,849 differentially methylated CpG sites corresponded with altered gene expression. Thus, it will be important to determine the effects of the extensive changes in DNA methylation profiles on genome integrity, nuclear architecture and nuclear matrix attachment, and their role in cellular senescence in future studies. It would also be important to analyse the roles of various histone modifications in senescence-associated gene expression regulation [47].

In summary, senescence in WI-38 cells was associated with the downregulation of DNMT1 and the global DNA hypomethylation. At the level of specific CpG sites, two-thirds of the differentially methylated sites were hypomethylated. However, differential DNA methylation did not correspond with differential gene expression in the majority of cases, suggesting that differential DNA methylation may affect the establishment of senescence through other mechanisms than transcriptional regulation. Further studies are needed to shed more light on the possible mechanisms involved and dissect the precise nature and biological repercussions of DNA methylation changes in senescence. Our datasets can be used as an important roadmap for the future analysis of the roles of DNA methylation in senescence.

## METHODS

### Cell culture

Human foetal lung fibroblasts (WI-38, ATCC, CCL-75TM) were maintained in HyClone minimum essential medium (MEM) Alpha Modification (ThermoScientific) containing 10% (v/v) foetal bovine serum (FBS) (Gibco) in a humidified Forma Steri-Cycle CO_2_ Incubator (ThermoScientific) at 37°C and 6% CO_2_. In order to compare the senescence-associated changes, three different population doubling (PD) levels of cells were used: PD 38 (proliferatively active), PD 47 (pre-senescent) and PD 54 (senescent), as described previously (manuscript 000141R1 in *AGING*).

### Cytosine extension assay

DNA was isolated from cells from two 10-cm cell culture plates per sample and three samples per PD level using the DNeasy® Blood and Tissue Kit (Qiagen) and a cytosine extension assay was performed as previously described [48]. A PerkinElmer Liquid Scintillation Analyzer Tri-Carb 2910 TR was used to measure CPM values.

### DNA methylation profiling

Genomic DNA (gDNA) was isolated from cells using the DNeasy^®^ Blood and Tissue Kit (Qiagen). Three samples per PD level were used, with each sample consisting of cells harvested from two 10 cm cell culture plates. 500 ng of DNA was used to determine DNA methylation profiles on Illumina^®^ HumanMethylation27 BeadChips, according to the manufacturer's protocol. Illumina^®^ GenomeStudio software was used for the determination of beta values (percentage of methylation of specific CpG sites). Differential DNA methylation analyses were performed using an Illumina Custom model, which produced Diff Scores as a measure of significance. Diff Scores of −13/13 were set as cut-off for significance.

### Functional classification

Functional classification of genes was done using several different softwares: FunNet Transcriptional Networks Analysis^1^], g:Profiler [49, 50], and DAVID Bioninformatics Resources 6.7 [51, 52] and compared with information on the genes available from the Genecards database^2^ [53]. All differentially methylated CpG sites were counted individually.

### Western immunoblotting

Protein was extracted by sonication of cells in 100 µL cold 1% SDS containing protease inhibitor (Roche). 40 µg of protein was separated by SDS-PAGE in slab gels of 6% (DNMT1) or 10% (MAT2A) polyacrylamide and transferred to Amersham Hybond-P polyvinylidene difluoride (PVDF) membranes (Amersham Biosciences). The membranes were incubated with primary antibodies overnight at 4°C (DNMT1 [Abcam], MAT2A [Abcam], GAPDH [Santa Cruz]). Antibody binding was revealed by incubation with horseradish peroxidase-conjugated secondary antibodies followed by ECL Plus immuno-blotting detection system (Amersham Biosciences). Chemiluminescence was detected using a FluorChem HD2 camera with FluorChem software (Cell Biosciences). The signals were quantified using NIH Image J64 software and normalized relative to GAPDH.

### Statistical analysis

All experiments, except the Illumina^®^ BeadChip determination of the DNA methylation pattern, included three biological replications, and statistically significant differences were determined by ANOVA (p < 0.05). For the Illumina^®^ HumanMethylation27 BeadChip, three replicates per population doubling level were studied and significant difference in site-specific methylation levels were determined as described above.

## SUPPLEMENTARY TABLE



## Footnotes

1 www.funnet.info

2 www.genecards.org
